# Genomic Characterization of Recent Chicken Anemia Virus Isolates in China

**DOI:** 10.3389/fmicb.2017.00401

**Published:** 2017-03-10

**Authors:** Yang Li, Lichun Fang, Shuai Cui, Jiayuan Fu, Xiaohan Li, Huanmin Zhang, Zhizhong Cui, Shuang Chang, Weifeng Shi, Peng Zhao

**Affiliations:** ^1^College of Veterinary Medicine, Shandong Agricultural UniversityTai’an, China; ^2^Avian Disease and Oncology Laboratory, Agricultural Research Service, United States Department of AgricultureEast Lansing, MI, USA; ^3^Institute of Pathogen Biology, Taishan Medical CollegeTai’an, China

**Keywords:** chicken anemia virus (CAV), genetic analysis, mammalian, phylogenetic analysis, recombination

## Abstract

Chicken anemia virus (CAV) causes diseases in young chickens, which include increased pathogenicity of secondary infectious agents, generalized lymphoid depletion, and immunodepression. In the present study, we have identified 22 CAV strains isolated from several commercial chicken farms in Northern China during 2014–2015. In addition, two CAVs were also isolated from stray mouse and dog feces, respectively. To our knowledge, this is the first report of identification of CAV from mouse and dog feces. Phylogenetic analysis of 121 full-length CAV genome sequences showed that all available CAV could be classified into eight lineages, supported by phylogenetic trees estimated using different methods. Furthermore, the 24 novel CAV sequences scattered across different branches, lack of clear spatio-temporal distribution characterization. Analysis of the 450 amino acids of VP1 protein identified 33 amino acid substitutions that were specific for CAVs from northern China. Putative gene recombination events were also detected in the genomes of newly isolated CAVs. In particular, a putative recombinant event was detected in the CAV-Dog genome with high statistical support. In summary, we established a robust classification system for CAV, revealed additional genomic diversity of CAV, and therefore, warranted additional efforts to explore CAV genomics and epidemiology.

## Introduction

Chicken anemia virus (CAV) was first isolated in Japan in 1979 (Yuasa et al., 1979) and has been commonly identified, since then, in chicken populations worldwide (Rosenberger and Cloud, 1989; Zhou et al., 1996; Ducatez et al., 2005; Natesan et al., 2006; Craig et al., 2009; Kim et al., 2010; Zhang et al., 2013). CAV causes atrophy of bone marrow hematopoietic tissue and lymphoid tissues (e.g., thymus) in young chickens, which leads to severe anemia and immunosuppression (Taniguchi et al., 1983). CAV spreads both horizontally and vertically (Jørgensen et al., 1995; Todd, 2000).

Chicken anemia virus currently belongs to the Gyrovirus genus, *Anelloviridae* family (Rosario et al., 2017). The CAV genome consists of a single molecule of circular (covalently closed end) single-stranded ambisense or negative sense (genus Gyrovirus) DNA (Ducatez et al., 2005), encoding peptides of 51.6 (VP1), 24 (VP2), and 13.6 (VP3) kDa, respectively (Noteborn et al., 1991). VP1 is the major viral structural protein, and is also associated with viral replication, cell infection ability and virulence (Renshaw et al., 1996; Yamaguchi et al., 2001; Todd et al., 2002). VP2 is a scaffolding protein, and both VP1 and VP2 can induce neutralizing antibodies (Koch et al., 1995). VP3 is a non-structural protein named as apoptin. The amino acid composition of CAV is very conservative, with only VP1 displaying significant variability in certain regions (e.g., amino acid positions139∼151) (Schat, 2003).

Chicken anemia virus has been detected in stray cat and human feces (Zhang et al., 2012, 2014). The molecular epidemiology of CAV has also been previously described. Zhang et al. (2013) analyzed 37 complete CAV genomes and classified them into two major groups. CAV was also subdivided into five groups, A–E, based on analysis of partial genomic sequences. By contrast, CAV was designated into four major clades, A–D, in an independent research (Eltahir et al., 2011a). However, no consistent classification of CAV has been established and the previous classification systems suffered from two major criticisms (Eltahir et al., 2011b; Zhang et al., 2013). First, the bootstrap values to support such classifications were extremely low. Second, several groups described in previous studies were not monophyletic.

In the present study, we described the characteristics of 24 novel strains of full-length CAV genome sequences, including two strains isolated from mice and dog feces. In addition, we analyzed 121 full-length CAV genome sequences using various phylogenetic methods and explored the potential association between genetic divergence of CAV and sampling dates. Based on the most comprehensive phylogenetic analysis to date, we proposed a more robust classification of CAV, which consisted of eight major lineages.

## Materials and Methods

### Clinical Samples Collection

A total of 70 liver and 45 bone marrow samples were collected from different commercial broiler chicken flocks, which showed clinical signs including thymus atrophy, pale bone marrow, swollen and discolored liver in seven provinces (Shandong, Hebei, Jiangsu, Liaoning, Henan, Neimenggu, and Beijing) of China during 2014–2015. Fecal samples from 42 stray dogs and 50 mice were collected from Taian, Shandong Province. All samples were stored at -80°C.

### DNA Isolation and CAV Detection

Total DNA was individually extracted from each of 207 suspected CAV positive samples using a commercial TIANamp Genomic DNA Kit (Taigen Biotechnology Co., Ltd., China) following the manufacturer’s instructions. A pair of PCR primers (forward: 5′-GCATTCCGAGTGGTTACTATTCC-3′; reverse: 5′-CGTCTTGCCATCTTACAGTCTTAT-3′) was designed based on the Cux-1 sequence (accession no. M55918) to detect CAV using DNAStar 6.0 (Madison, WI, USA). The amplicon was expected for 982 bp in length. PCR was performed using the TaKaRa Ex Taq kit (TaKaRa Bio, Inc., Shiga, Japan) according to the manufacturer’s instructions.

### Virus Isolation

Twenty-four PCR positive samples were propagated in Marek’s disease virus transformed MSB-1 cell line as previously described (Goryo et al., 1987). Briefly, 0.5 ml of the filtered sample supernatant was first mixed with the MSB-1 cell pellet, which was then re-suspended in 0.5 ml of 1640 medium, followed by incubation at 37°C for 1 h. Then, 5 ml of 1640 medium were added to the mixture, and cells were suspended followed by incubation at 37°C for 3 days. 1 ml cell suspension was transferred to 4 ml of fresh 1640 medium and incubated. The blind passage was carried out every 3 days, until the appearance of cytopathogenic effects (CPE). Then the media of MSB1 cells were collected and repeatedly frozen and thawed three times. The cells were repeatedly passaged until a high virus titer was obtained, and the presence of virus was detected by PCR.

### Amplification, Cloning, and Sequencing of the CAV Genomes

Three additional pairs of PCR primers were designed using DNAStar6.0 based on published CAV sequences in GenBank, which were expected to generate amplicons of 843, 989, and 802 bp each in length encompassing the entire CAV genome (Supplementary Table S1). The total DNA was extracted from the cultured cell supernatant of the 24 CAV positive samples. PCR were performed in a 25 μL reaction volume containing 0.5 μL forward primer, 0.5 μL reverse primer, 1 μL DNA, 2.5 μL 10 × PCR buffer (Mg^2+^), 2 μL dNTPs (2.5 mM), 0.5 μL rTaq DNA polymerase (TaKaRa Biotechnology Co. Ltd., Dalian, China), and 18 μL double-distilled H_2_O. PCR cycle conditions were 95°C for 5 min for pre-denaturation, followed by 30 cycles of 95°C for 30 s, 55°C for 50 s, and 72°C for 60 s; a final extension step was set at 72°C for 10 min prior to termination of the reaction at 4°C. The PCR products were analyzed by 1% agarose gel electrophoresis.

All PCR for each sample were performed in duplicate. Both positive and negative controls were included for each batch of PCR. The PCR products were analyzed in 1% agarose gels stained with ethidium bromide. The amplicons were purified with a Gel Band Purification Kit (Omega Bio-Tek, USA), cloned into the pMD-18T vector (TaKaRa Bio Inc., Japan), and the sequenced in triplicate on an ABI 3730 Sanger-based genetic analyzer (Carlsbad, CA, USA).

### Sequence Alignment and Phylogenetic Analysis

DNA sequences of the cloned CAV amplicons were assembled and, along with 97 full-length CAV genome sequences downloaded from GenBank, were analyzed using DNAStar version 6.0 (DNAStar Inc., Madison, WI, USA). Multiple sequence alignment was performed using Muscle (Edgar, 2004). Sequence identity was estimated using SDT (Muhire et al., 2014).

Phylogenetic analysis of the 24 complete CAV genomes and 97 reference sequences obtained from GenBank was performed using different methods. Maximum likelihood (ML) analysis was performed in RAxML using the full-length genomes and the VP1 regions, respectively (Stamatakis, 2006). The GTRGAMMAI model was designated as the nucleotide substitution model, and the robustness of the ML tree was evaluated by a bootstrap analysis of 1000 replicates. A Bayesian tree was estimated using MrBayes using the General Time Reversible (GTR) nucleotide substitution model (Huelsenbeck and Ronquist, 2001). Four independent chains were run for 50 million steps with trees and parameters sampled every 5000 steps, and the first 10% were removed as burn-in. To further investigate the relationship between genetic divergence and collection times of CAV, we explored the temporal structure of the CAV dataset using TempEst, and the ML and Bayesian trees estimated in the previous step were used as the input (Rambaut et al., 2016). However, the correlation coefficient, *R*^2^, obtained from TempEst was close to zero, failing to establish the strong association between genetic distances and sampling dates. Therefore, we did not estimate the time-scaled trees for the CAV dataset. In addition, the TempEst analysis revealed that the two Australian strains (accession nos: AF227982 and EF683159) were outliers and therefore they were used as outgroup sequences.

### Detection of Putative Recombination Events

Putative recombination events within each of the CAV genomes were detected using the Recombination Detection Program 4 (RDP4) (Martin, 2009). Other detection methods, including RDP, GENECONV, BootScan, MaxChi, Chimera, SiScan, Phyl-Pro, LARD, and 3Seq, were also employed for comparison purpose (Martin et al., 2005a,b). Only recombination events supported by no fewer than six independent methods were regarded as positive. These putative recombinants events were further confirmed and visualized using SimPlot (Lole et al., 1999).

### Ethics Statement

The use of animals for this study was approved by the Animal Welfare Committee of our Institute. All of the experimental animals of this study were cared for and maintained throughout of the experiments strictly following the ethics and biosecurity guidelines approved by the Institutional Animal Care and Use Committee of Shandong Agricultural University.

## Results

### Identification of CAV

A total of 24 CAV samples were cultured and blindly passaged in MDCC-MSB1 cells. The cell supernatant was harvested 3 days later and the presence of CAV was confirmed by PCR. All 24 CAV isolates were confirmed positive (**Table 1**), which included one isolate from Beijing (in 2014) and Neimenggu (in 2015) each, two from Liaoning province (in 2014), Hebei province (in 2014 and 2015), Henan province (in 2014 and 2015) each, three from Jiangsu province (in 2015), eleven from Shandong province (in 2015), one from stray mice feces and one from dog feces.

**Table 1:** Details of the 24 novel CIAV genome sequences.

Accession NO.	Strain name	Year	Province	Host	Length	Biological sample
KU641013	LN1401	2014	Liaoning	Chicken	2298 bp	Liver
KU645511	LN1402	2014	Liaoning	Chicken	2298 bp	Liver
KU645514	HB1404	2014	Hebei	Chicken	2298 bp	Liver
KU645516	HB1517	2015	Hebei	Chicken	2298 bp	Liver
KU645518	JS1501	2015	Jiangsu	Chicken	2298 bp	Bone marrow
KU645508	JS1502	2015	Jiangsu	Chicken	2298 bp	Bone marrow
KU645509	JS1503	2015	Jiangsu	Chicken	2298 bp	Bone marrow
KU645520	HN1405	2014	Henan	Chicken	2298 bp	Liver
KU645512	HN1504	2015	Henan	Chicken	2298 bp	Liver
KU645517	SD1513	2015	Neimenggu	Chicken	2298 bp	Bone marrow
KU645513	BJ1406	2014	Beijing	Chicken	2298 bp	Bone marrow
KU641014	JN1503	2015	Shandong	Chicken	2298 bp	Bone marrow
KU645523	SD1505	2015	Shandong	Chicken	2298 bp	Liver
KU645507	SD1507	2015	Shandong	Chicken	2298 bp	Liver
KU645519	SD1508	2015	Shandong	Chicken	2298 bp	Bone marrow
KU645510	SD1509	2015	Shandong	Chicken	2298 bp	Bone marrow
KU598851	SD1510	2015	Shandong	Chicken	2298 bp	Bone marrow
KU641015	SD1511	2015	Shandong	Chicken	2298 bp	Liver
KU645506	SD1512	2015	Shandong	Chicken	2298 bp	Liver
KU645521	SD1514	2015	Shandong	Chicken	2298 bp	Liver
KU645515	SD1515	2015	Shandong	Chicken	2298 bp	Liver
KU645522	SD1518	2015	Shandong	Chicken	2298 bp	Liver
KU645524	CAV-Dog	2015	Shandong	Dog	2298 bp	Feces
KU645525	CAV-Mouse	2015	Shandong	Mouse	2298 bp	Feces

### Sequence Identities of the 24 CAV Strains

Full-length genome sequences of 24 CAV isolates were characterized comparative genomics. The nucleotide sequence identities between the 22 CAV isolate genomes and 97 CAV isolates retrieved from GenBank ranged between 94.8 and 99.3%. The highest identity (99.3%) was found between the chicken CAV isolates BJ1406 and SC-MZ (accession no. KM496308, isolated from Sichuan, China, in 2014). The lowest identity (94.8%) was found between the chicken CAV isolates HB1517 and 3711 (accession no. EF683159, isolated from Australia in 2007).

The nucleotide sequence identities between the dog derived CAV isolate and the 119 CAV-chicken isolates (97 from GenBank and 22 from this study) ranged from 94.9% (the isolate 3711, accession no. EF683159) to 98.3% (the isolate SD24, accession no. AY999018, isolated in Tianjin of China, in 2005). The sequence identities between the mouse feces derived isolate and chicken isolates ranged 95.0% (the isolate 14, accession no. KJ728824) to 99.6% (the isolate JN1503, accession no. KU641014, isolated from Shandong, China in 2015). Both of the two mammalian feces derived isolates had very low identities to the strain, 3711 (accession no. EF683159).

### Molecular Characterization of the 24 CAV Genomes

The complete VP1, VP2, and VP3 gene sequences of the 24 CAV strains were 1350, 651 and 366 nucleotides in length, respectively. No nucleotide insertions or deletions were identified compared to the 97 released worldwide CAV genomes. Analysis of the 450 amino acids of VP1 identified 33 amino acid substitutions that were specific for chicken CAVs isolated in northern China. Nucleotide sequence analyses of the chicken CAV isolates led to identifications of 27 and 12 single nucleotide polymorphisms in VP2 and VP3 gene regions, respectively, which consequently led to 14 and 5 amino acid substitutions in VP2 and VP3, respectively. No nucleotide substitution was identified in the VP2 and VP3 genes of the dog and mouse feces derived strains.

**FIGURE 1:**
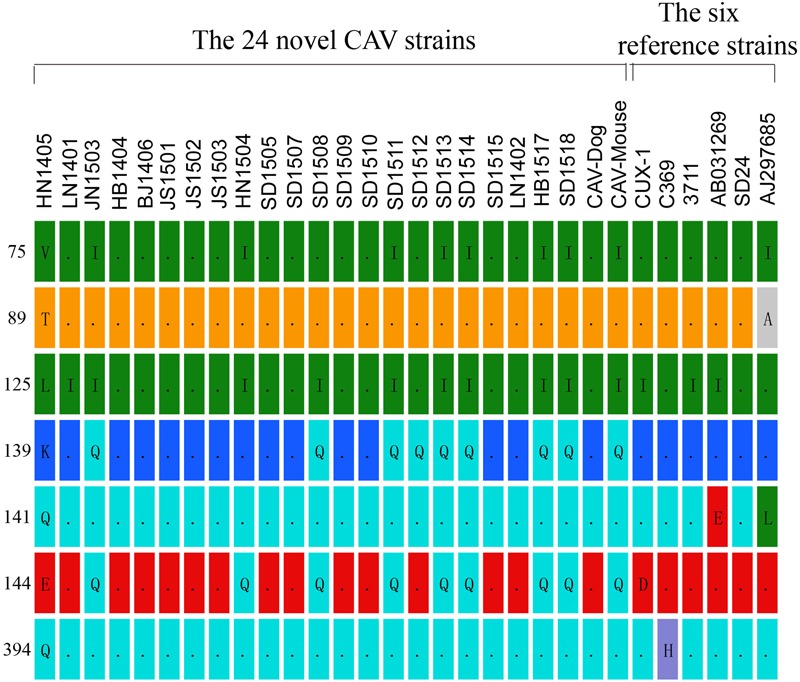
**Amino acid substitutions observed among the 24 novel CAV strains in contrast to six reference isolates (Cux-1, 369, 3711, AB03129, SD24, AJ297685)**.

In the hypervariable region (from position 139 to 151)of the VP1 protein, positions 139 and 144 have been reported to play an important role in viral growth and spread, and Q139 and/or Q144 were associated with decreased spread (Renshaw et al., 1996). Among the genomes of the 24 CAV strains, 8 out of the 24 genomes carried both Q139 and Q144, one (HN1504) carried only Q144, and one (SD1512) carried Q139, suggesting that these strains might have relatively low rates of growth and spread (**Figure 1**). The remaining 14 CAVs contained residues K139 and E144. In addition, residue 394 of the VP1 protein was a major genetic determinant of virulence, with glutamine (Q) and histidine (H) representing high and low pathogenicity, respectively (Yamaguchi et al., 2001; Todd et al., 2002). All of the CAV strains isolated from northern China possessed a glutamine at position 394, suggesting that those strains might be highly pathogenic (**Figure 1**). Previous studies have also showed that if amino acid substitutions occurred simultaneously at positions 75, 89, 125, 141, and 144 of the VP1 protein, CAV would not cause anemia, white bone marrow and thymus atrophy (Todd et al., 2002). In this study, it was observed that valine (Val) and leucine (Leu) at positions 75 and 125 of the VP1 protein were replaced by isoleucine (Ile) in seven chicken CAV strains and the CAV-Mouse strain. However, the simultaneous amino acid substitutions at the five positions were not observed among the 24 novel sequences (**Figure 1**).

We then systematically compared the amino acid polymorphisms of the four mammalian-origin CAVs and representative chicken CAV strains (Supplementary Figure S1). No amino acid substitutions were identified to be commonly possessed only by the mammalian feces-origin CAVs (Supplementary Figure S1). In addition, all of the 11 amino acid sites examined in the mammalian-origin CAVs displayed amino acid polymorphism (two different amino acids at a single site) and these amino acids could be found in chicken CAV strains.

### Phylogenetic Analysis of 121 Complete CAV Genome Sequences

**FIGURE 2:**
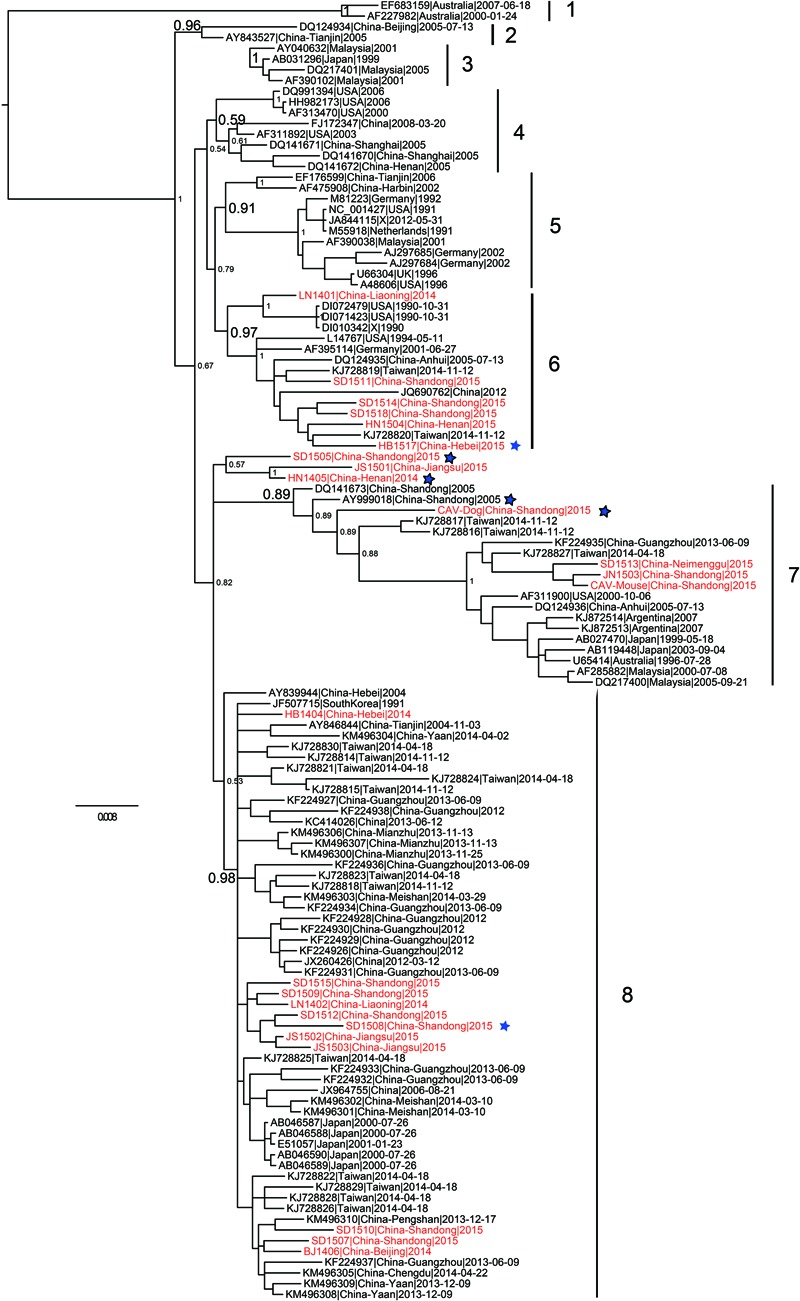
**Phylogenetic tree of 121 CAV full-length genome sequences estimated using MrBayes.** The 24 novel Chinese isolates are shown in red. The six potential recombinant isolates are marked with a blue five-pointed star, respectively.

Phylogenetic analysis of 121 (24 from the present study and 97 from public database) full-length CAV genome sequences were performed using different methods. The Bayesian inference showed that CAV could be classified into eight major lineages, 1–8, supported by the topology and the high posterior probability (generally > 0.89) (**Figure 2**). This classification was also supported by the phylogenetic trees estimated using the non-recombinant CAV genome sequences (Supplementary Figure S2) and using the ML method (Supplementary Figure S3), suggesting it was robust, although the bootstrap values of some lineages in the ML tree were not high (Supplementary Figure S3). In particular, this classification was not fully consistent with previous ones established using just a few number of CAV genomes. Our results revealed that the phylogenetic relationships of CAV were more complicated than those previously described.

In addition, the 24 novel CAV sequences were scattered across different branches, without clear spatio-temporal distribution characteristics (**Figure 2**). Six strains fell within lineage 6, with a few isolates from The United States and Germany. Four strains were clustered into lineage 7 and 8 into lineage 8. Lineage 7 consisted of worldwide CAVs, while lineage 8 just included strains from Eastern Asia. In addition, the remaining three novel CAV sequences were not classified into any lineage in the proposed classification (**Figure 2**). Two of them were subsequently detected to be potential recombinants, which might account for their heterogeneous genomic composition.

As for the two novel mammalian feces-derived isolates, both of them fell within lineage 7. The mouse feces derived strain and the two chicken strains (JN1503 and SD1513) were placed in a small cluster with strong bootstrap support. In contrast, the dog feces derived isolate was closely related to SD24 (an isolate from Shandong in 2005, accession no. AY999018) and two isolates from Taiwan.

### Detection of Putative Recombination Events

**FIGURE 3:**
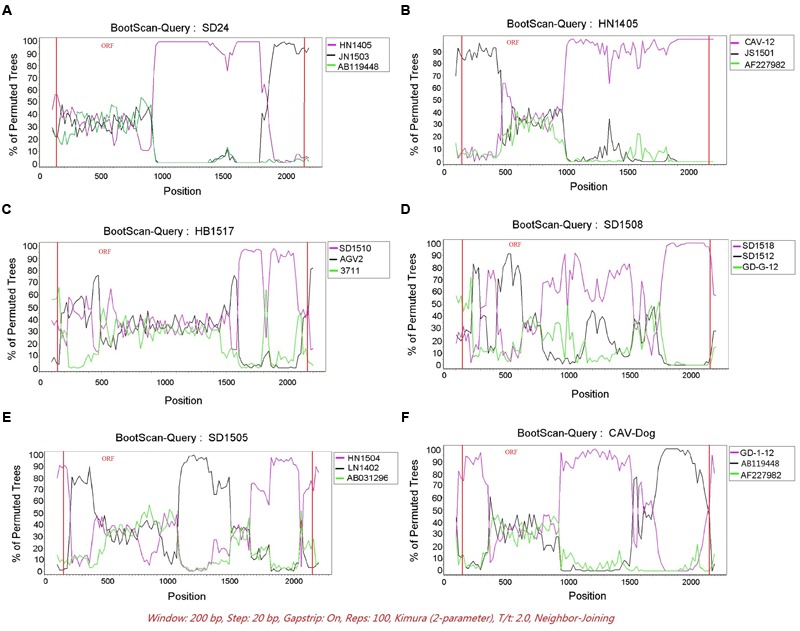
**The Bootscanning analysis of the six putative recombinants.** SIMPLOT recombination analysis. **(A–F)** Bootscanning analysis performed with the six putative recombinants as query sequences with siding window of 200bp and a step size of 20 bp.

The RDP was run to identify putative recombinant events of the 121 CAV genome sequences. Only the recombination events supported by at least five independent detection methods were regarded as reliable. A total of six novel recombination events were identified (**Figure 3**), with one in lineage 6, two in lineage 7, one in lineage 8 and two unclassified (**Figure 2**). Breakpoints and potential parents of the recombinants were also identified. For example, for the strain SD24, the breakpoints were detected to be approximately located at positions 1684 and 57 (**Figure 3A**), which was from the major parent of HN1405 and the minor parent of JN1503. In particular, a recombinant event was detected in the dog feces derived -CAV strain, which was from the major parent of GD-1-12 (isolated from Guangdong, China) and the minor parent of AB119448 (isolated from Gifu, Japan).

## Discussion

Chicken anemia virus causes severe anemia in newly hatched chickens and destruction of erythroblastoid cells in bone marrow and thymocytes in thymic cortex (Kato et al., 1995). In China, CAV was first isolated in 1996 (Zhou et al., 1996). Apart from chickens, CAV has also been identified from stray cat feces, human feces, blood and skin (Zhang et al., 2012, 2014). In this study, we characterized the full-length sequences of 24 novel CAV genomes of CAV strains isolated from five (Liaoning, Hebei, Neimenggu, Beijing, and Shandong) provinces in Northern China and two (Jiangsu and Henan) in Southern China. In particular, two CAVs were isolated from stray mice and dog feces, respectively. However, there is no evidence that the virus could replicate in mice and dogs.

Comparison between the genomes of the 24 CAV strains with the 97 reported CAV genomes, we identified unique nucleotide and amino acid substitutions in the 24 novel CAV genomes. These novel substitutions, interestingly, were spotted only in the VP1 region. This is in good agreement with reports that the N-terminal half of VP3 and the N-terminal three quarters of VP2 are well conserved to sustain an essential function of these proteins (Farkas et al., 1996). It was also speculated that the amino acid at position 394 of the VP1 protein serves as a major genetic determinant of virulence (Yamaguchi et al., 2001). All of these newly sequenced 24 Chinese strains possessed Q at position 394, which indicated that they might be highly pathogenic. The amino acids at positions 139 and 144 play an important role in virus growth and spread (Renshaw et al., 1996). Eight out of the 24 novel strains possessed both Q139 and Q144; one (SD1512) had 139Q, and another (HN1504) only had 144Q, which suggested these strains might have low rates of growth and spread capacity.

The phylogeny constructed using the 121 full-length CAV genomes clearly showed that the CAV genomes lacked of spatio-temporal distribution characteristics and the 24 novel CAV genomes were interspersed across the branches (**Figure 2**). The mouse feces associated CAV strain was closely related to chicken isolates from Shandong province, and the dog feces associated CAV strain was closely related to chicken isolates from Taiwan, suggesting the potential origin of the strain from eating chicken meat. Based on the most comprehensive phylogenetic analysis performed so far, we proposed a robust classification for CAV, which was supported by different phylogenetic methods. However, this phylogeny outcome is poorly in agreement with reports in literature, and may indicate the existence of further complexity CAV on genome, pathology, and epidemiology, which warrants additional in depth investigations.

It should also be noted that the TempEst analysis revealed little temporal structure in the CAV dataset and failed to establish the strong association between the genetic divergence of CAV and sampling dates (see Materials and Methods). Therefore, based on the current evidence, it is still unfeasible to accurately trace the origin and explore the molecular evolutionary patterns of CAV as results from the time-scaled phylogenetic analyses may not be reliable. To this end, more worldwide surveillance data of CAV is needed.

DNA recombination plays an important role in evolution of many viruses, such as hepatitis B virus (HBV) and human enteroviruses, and is essential in generating and maintaining genetic diversity of viruses. As a DNA virus, CAV has rarely been found to undergo gene recombination. However, Eltahir et al. (2011a) reported genetic recombination of CAV, which was identified in the coding regions. Zhang et al. (2013) also reported CAV recombination events occurred in non-coding regions. From this study, we reported a total of six potential recombination events, which once again highlighted the important role of genetic recombination in shaping the genetic diversity of CAV.

In summary, we described 24 novel full-length CAV genomes, including two from mouse and dog. We revealed additional genomic diversity of CAV. The findings warrant additional efforts to explore CAV genomics and epidemiology, which ought to eventually lead to better understanding on the evolution of CAV.

## Author Contributions

YL conceived and performed the experiments, analyzed the data, and drafted the manuscript. PZ, WS, and SCu supervised the project and edited the manuscript. LF and SCu conducted part of the experiments. SCh, HZ, and JF analyzed part of the data. XL and ZC provided important suggestions.

## Conflict of Interest Statement

The authors declare that the research was conducted in the absence of any commercial or financial relationships that could be construed as a potential conflict of interest.
