# Supplementary material for: Genomic Characterization of Recent Chicken Anemia Virus Isolates in China
Source: Front Microbiol. 2017 Mar 10;8:401. doi: 10.3389/fmicb.2017.00401 (PMC5344997; doi:10.3389/fmicb.2017.00401)
Supplement: Supplementary file 1 [file Data_Sheet_1.pdf]

Supplementary Information

Title: Genomic Characterization of Recent Chicken Anemia Virus Isolates in China

Author: Yang Li, Lichun Fang, Jiayuan Fu, Shuai Cui, Xiaohan Li,

Huanmin Zhang, Zhizhong Cui, Shuang Chang, Weifeng Shi, Peng Zhao

Supplementary Table S1 and Figure S1-3

Table S1. Primers used for genome amplification.

Fig. S1: The amino acid polymorphisms of the mammalian and chicken CAV sequences. The first four strains were mammalian feces-origin CAVs (cat, human, dog, mouse). Ten chicken CAV strains were selected as references.

Fig. S2: Phylogenetic tree of 115 non-recombinant CAV full-length genome sequences estimated using MrBayes. In this analysis, the six potential recombinant sequences were removed. The 18 novel Chinese isolates are shown in red.

Fig. S3: The Maximum Likelihood phylogenetic analysis of 121 CAV full-length genome sequences using RAxML. The 24 novel Chinese isolates are shown in red. The six potential recombinant isolates are marked with a blue five-pointed star, respectively.

Supplementary Table S1. Primers used for genome amplification.

Primers	Sequence	Product length
F1	5’- GCATTCCGAGTGGTTACTATTCC-3’	843bp
R1	5’- CGTCTTGCCATCTTACAGTCTTAT-3’	
F2	5’- CGAGTACAGGGTAAGCGAGCTAAA-3’	989bp
R2	5’- TGCTATTCATGCAGCGGACTT-3’	
F3	5’- ACGAGCAACAGTACCCTGCTAT-3’	802bp
R3	5’- CTGTACATGCTCCACTCGTT-3’	

Fig. S1

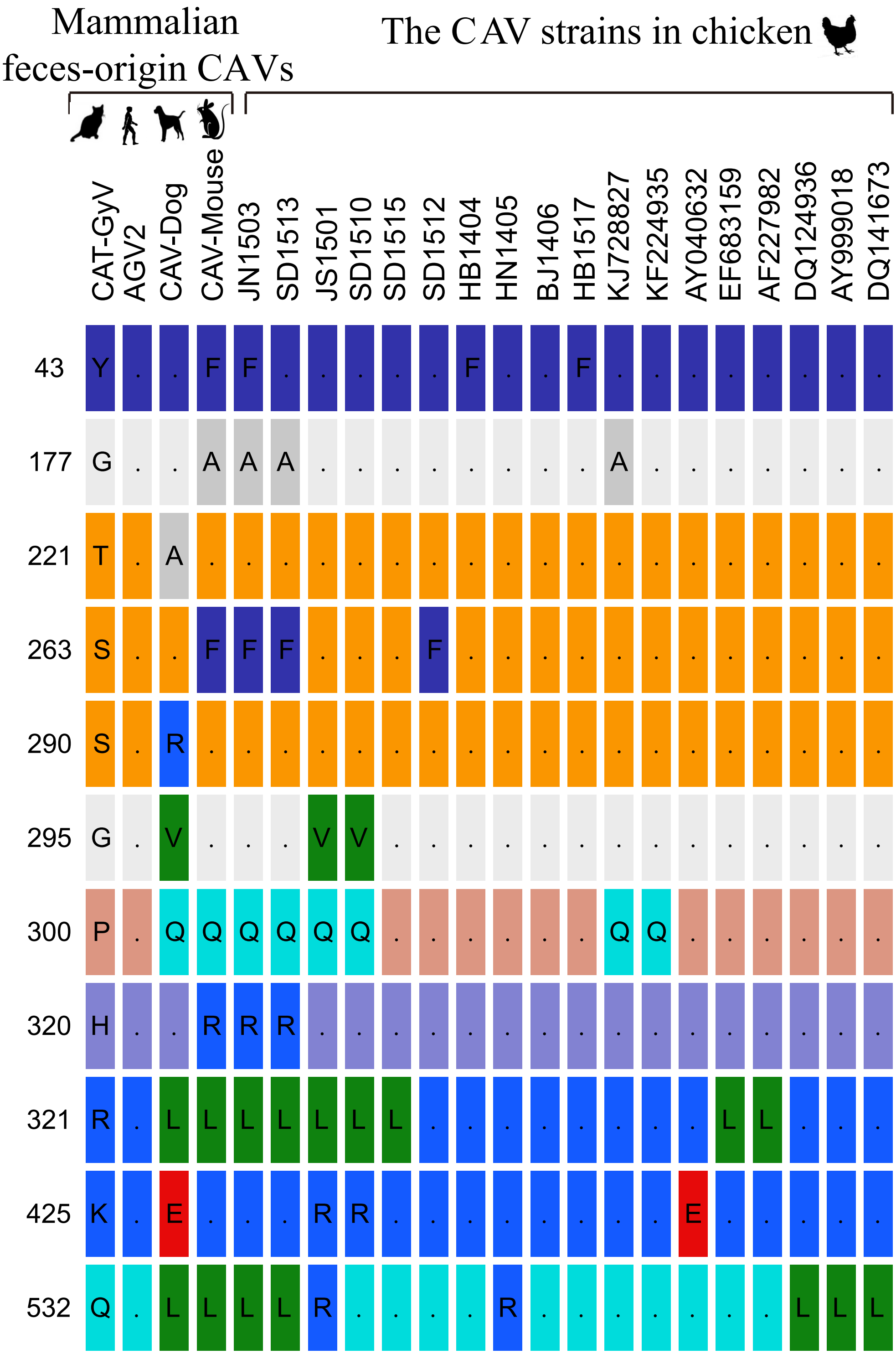

Mammalian feces-origin CAVs
The CAV strains in chicken
CAT-GyV
AGV2
CAV-Dog
CAV-Mouse
JN1503
SD1513
JS1501
SD1510
SD1515
SD1512
HB1404
HN1405
BJ1406
HB1517
KJ728827
KF224935
AY040632
EF683159
AF227982
DQ124936
AY999018
DQ141673
43 Y . . F F . . . . . F . . F . . . .
177 G . . A A A . . . . A . . . .
221 T . A . . . .
263 S . . F F F . . . F . . . .
290 S . R . . . .
295 G . V . . . V V . . . .
300 P . Q Q Q Q Q Q . . . . . . Q Q . . . . . .
320 H . . R R R . . . .
321 R . L L L L L L L . . . . L L . . .
425 K . E . . . R R . . . . E . . . . .
532 Q . L L L L R . . . . R . . . . L L L

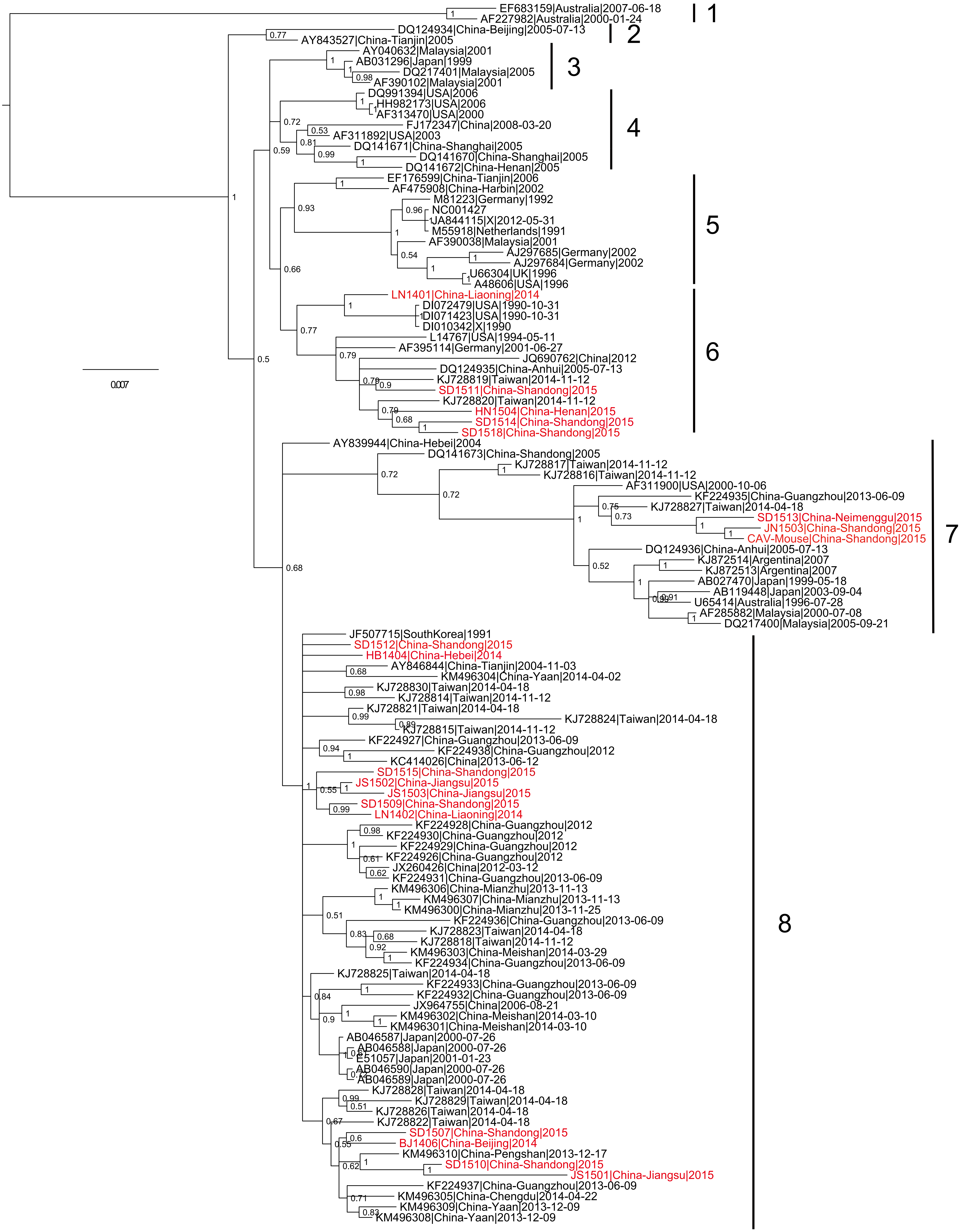

EF683159|Australia|2007-06-18
AF227982|Australia|2000-01-24
1
DQ124934|China-Beijing|2005-07-13
AY843527|China-Tianjin|2005
2
AY040632|Malaysia|2001
AB031296|Japan|1999
DQ217401|Malaysia|2005
AF390102|Malaysia|2001
3
DQ991394|USA|2006
HH982173|USA|2006
AF313470|USA|2000
FJ172347|China|2008-03-20
AF311892|USA|2003
DQ141671|China-Shanghai|2005
DQ141670|China-Shanghai|2005
DQ141672|China-Henan|2005
4
EF176599|China-Tianjin|2006
AF475908|China-Harbin|2002
M81223|Germany|1992
NC001427
JA844115|X|2012-05-31
M55918|Netherlands|1991
AF390038|Malaysia|2001
AJ297685|Germany|2002
AJ297684|Germany|2002
U66304|UK|1996
A48606|USA|1996
5
LN1401|China-Liaoning|2014
DI072479|USA|1990-10-31
DI071423|USA|1990-10-31
DI010342|X|1990
L14767|USA|1994-05-11
AF395114|Germany|2001-06-27
JQ690762|China|2012
DQ124935|China-Anhui|2005-07-13
KJ728819|Taiwan|2014-11-12
SD1511|China-Shandong|2015
KJ728820|Taiwan|2014-11-12
HN1504|China-Henan|2015
SD1514|China-Shandong|2015
SD1518|China-Shandong|2015
6
0.007
AY839944|China-Hebei|2004
DQ141673|China-Shandong|2005
KJ728817|Taiwan|2014-11-12
KJ728816|Taiwan|2014-11-12
AF311900|USA|2000-10-06
KF224935|China-Guangzhou|2013-06-09
KJ728827|Taiwan|2014-04-18
SD1513|China-Neimenggu|2015
JN1503|China-Shandong|2015
CAV-Mouse|China-Shandong|2015
DQ124936|China-Anhui|2005-07-13
KJ872514|Argentina|2007
KJ872513|Argentina|2007
AB027470|Japan|1999-05-18
AB119448|Japan|2003-09-04
U65414|Australia|1996-07-28
AF285882|Malaysia|2000-07-08
DQ217400|Malaysia|2005-09-21
7
JF507715|SouthKorea|1991
SD1512|China-Shandong|2015
HB1404|China-Hebei|2014
AY846844|China-Tianjin|2004-11-03
KM496304|China-Yaan|2014-04-02
KJ728830|Taiwan|2014-04-18
KJ728814|Taiwan|2014-11-12
KJ728821|Taiwan|2014-04-18
KJ728824|Taiwan|2014-04-18
KJ728815|Taiwan|2014-11-12
KF224927|China-Guangzhou|2013-06-09
KF224938|China-Guangzhou|2012
KC414026|China|2013-06-12
SD1515|China-Shandong|2015
JS1502|China-Jiangsu|2015
JS1503|China-Jiangsu|2015
SD1509|China-Shandong|2015
LN1402|China-Liaoning|2014
KF224928|China-Guangzhou|2012
KF224930|China-Guangzhou|2012
KF224929|China-Guangzhou|2012
KF224926|China-Guangzhou|2012
JX260426|China|2012-03-12
KF224931|China-Guangzhou|2013-06-09
KM496306|China-Mianzhu|2013-11-13
KM496307|China-Mianzhu|2013-11-13
KM496300|China-Mianzhu|2013-11-25
KF224936|China-Guangzhou|2013-06-09
KJ728823|Taiwan|2014-04-18
KJ728818|Taiwan|2014-11-12
KM496303|China-Meishan|2014-03-29
KF224934|China-Guangzhou|2013-06-09
KJ728825|Taiwan|2014-04-18
KF224933|China-Guangzhou|2013-06-09
KF224932|China-Guangzhou|2013-06-09
JX964755|China|2006-08-21
KM496302|China-Meishan|2014-03-10
KM496301|China-Meishan|2014-03-10
AB046587|Japan|2000-07-26
AB046588|Japan|2000-07-26
E51057|Japan|2001-01-23
AB046590|Japan|2000-07-26
AB046589|Japan|2000-07-26
KJ728828|Taiwan|2014-04-18
KJ728829|Taiwan|2014-04-18
KJ728826|Taiwan|2014-04-18
KJ728822|Taiwan|2014-04-18
SD1507|China-Shandong|2015
BJ1406|China-Beijing|2014
KM496310|China-Pengshan|2013-12-17
SD1510|China-Shandong|2015
JS1501|China-Jiangsu|2015
KF224937|China-Guangzhou|2013-06-09
KM496305|China-Chengdu|2014-04-22
KM496309|China-Yaan|2013-12-09
KM496308|China-Yaan|2013-12-09
8

Fig. S3

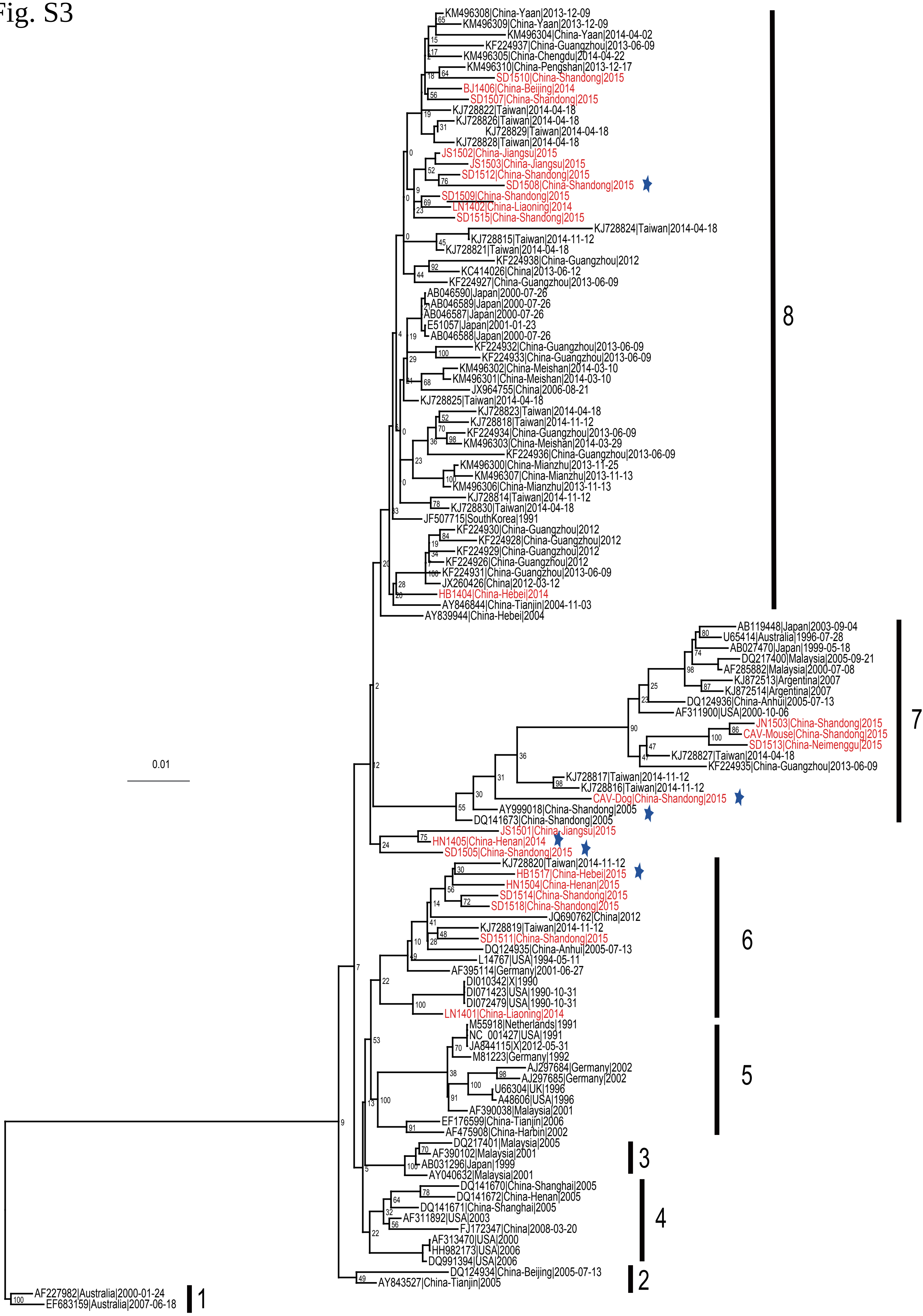
KM496308|China-Yaan|2013-12-09
KM496309|China-Yaan|2013-12-09
KM496304|China-Yaan|2014-04-02
KF224937|China-Guangzhou|2013-06-09
KM496305|China-Chengdu|2014-04-22
KM496310|China-Pengshan|2013-12-17
SD1510|China-Shandong|2015
BJ1406|China-Beijing|2014
SD1507|China-Shandong|2015
KJ728822|Taiwan|2014-04-18
KJ728826|Taiwan|2014-04-18
KJ728829|Taiwan|2014-04-18
KJ728828|Taiwan|2014-04-18
JS1502|China-Jiangsu|2015
JS1503|China-Jiangsu|2015
SD1512|China-Shandong|2015
SD1508|China-Shandong|2015
SD1509|China-Shandong|2015
LN1402|China-Liaoning|2014
SD1515|China-Shandong|2015
KJ728824|Taiwan|2014-04-18
KJ728815|Taiwan|2014-11-12
KJ728821|Taiwan|2014-04-18
KF224938|China-Guangzhou|2012
KC414026|China|2013-06-12
KF224927|China-Guangzhou|2013-06-09
AB046590|Japan|2000-07-26
AB046589|Japan|2000-07-26
AB046587|Japan|2000-07-26
E51057|Japan|2001-01-23
AB046588|Japan|2000-07-26
KF224932|China-Guangzhou|2013-06-09
KF224933|China-Guangzhou|2013-06-09
KM496302|China-Meishan|2014-03-10
KM496301|China-Meishan|2014-03-10
JX964755|China|2006-08-21
KJ728825|Taiwan|2014-04-18
KJ728823|Taiwan|2014-04-18
KJ728818|Taiwan|2014-11-12
KF224934|China-Guangzhou|2013-06-09
KM496303|China-Meishan|2014-03-29
KF224936|China-Guangzhou|2013-06-09
KM496300|China-Mianzhu|2013-11-25
KM496307|China-Mianzhu|2013-11-13
KM496306|China-Mianzhu|2013-11-13
KJ728814|Taiwan|2014-11-12
KJ728830|Taiwan|2014-04-18
JF507715|SouthKorea|1991
KF224930|China-Guangzhou|2012
KF224928|China-Guangzhou|2012
KF224929|China-Guangzhou|2012
KF224926|China-Guangzhou|2012
KF224931|China-Guangzhou|2013-06-09
JX260426|China|2012-03-12
HB1404|China-Hebei|2014
AY846844|China-Tianjin|2004-11-03
AY839944|China-Hebei|2004
8
AB119448|Japan|2003-09-04
U65414|Australia|1996-07-28
AB027470|Japan|1999-05-18
DQ217400|Malaysia|2005-09-21
AF285882|Malaysia|2000-07-08
KJ872513|Argentina|2007
KJ872514|Argentina|2007
DQ124936|China-Anhui|2005-07-13
AF311900|USA|2000-10-06
JN1503|China-Shandong|2015
CAV-Mouse|China-Shandong|2015
SD1513|China-Neimenggu|2015
KJ728827|Taiwan|2014-04-18
KF224935|China-Guangzhou|2013-06-09
KJ728817|Taiwan|2014-11-12
KJ728816|Taiwan|2014-11-12
CAV-Dog|China-Shandong|2015
AY999018|China-Shandong|2005
DQ141673|China-Shandong|2005
7
0.01
JS1501|China-Jiangsu|2015
HN1405|China-Henan|2014
SD1505|China-Shandong|2015
KJ728820|Taiwan|2014-11-12
HB1517|China-Hebei|2015
HN1504|China-Henan|2015
SD1514|China-Shandong|2015
SD1518|China-Shandong|2015
JQ690762|China|2012
KJ728819|Taiwan|2014-11-12
SD1511|China-Shandong|2015
DQ124935|China-Anhui|2005-07-13
L14767|USA|1994-05-11
AF395114|Germany|2001-06-27
DI010342|X|1990
DI071423|USA|1990-10-31
DI072479|USA|1990-10-31
LN1401|China-Liaoning|2014
6
M55918|Netherlands|1991
NC_001427|USA|1991
JA844115|X|2012-05-31
M81223|Germany|1992
AJ297684|Germany|2002
AJ297685|Germany|2002
U66304|UK|1996
A48606|USA|1996
AF390038|Malaysia|2001
EF176599|China-Tianjin|2006
AF475908|China-Harbin|2002
5
DQ217401|Malaysia|2005
AF390102|Malaysia|2001
AB031296|Japan|1999
AY040632|Malaysia|2001
3
DQ141670|China-Shanghai|2005
DQ141672|China-Henan|2005
DQ141671|China-Shanghai|2005
AF311892|USA|2003
FJ172347|China|2008-03-20
AF313470|USA|2000
HH982173|USA|2006
DQ991394|USA|2006
4
DQ124934|China-Beijing|2005-07-13
AY843527|China-Tianjin|2005
2
AF227982|Australia|2000-01-24
EF683159|Australia|2007-06-18
1
